# The PARTHENON Clinical Development Program: the Role of Ticagrelor in Patients with Atherothrombotic Disease

**DOI:** 10.1007/s10557-017-6749-7

**Published:** 2017-09-02

**Authors:** Paul P. Dobesh, Manesh Patel

**Affiliations:** 1 College of Pharmacy, University of Nebraska Medical Center, 986145 Nebraska Medical Center, Omaha, NE 68198-6145 USA; 2 Division of Cardiovascular Medicine, Duke University Medical Center, Duke Clinical Research Institute, Durham, DC USA

**Keywords:** Acute coronary syndrome, Cardiovascular disease, Parthenon, Ticagrelor

## Abstract

Although the rate of cardiovascular disease (CVD)-related mortality has declined over the last decade, it is still the leading cause of mortality in the USA, accounting for over 1.4 million deaths annually. In addition, total direct (primarily hospital admissions) and indirect costs of CVD in the US is over $316 billion annually and is expected to grow to over $918 billion by 2030. Much of the etiology of CVD is due to atherosclerosis and its thrombotic complications, and central to this is the role of platelets. Atherosclerosis is a systemic disease, with meaningful morbidity and mortality when present in the coronary, cerebral, or major peripheral arteries. The recommended antiplatelet therapy differs based on the vascular bed impacted, with the optimal antiplatelet therapy yet to be defined. The PARTHENON program is a series of completed and ongoing phase III clinical trials investigating the efficacy and safety of ticagrelor in atherosclerotic CVD in comparison with established antiplatelet therapy or placebo. The overall aim of the program is to determine if more potent antiplatelet therapy, with different pharmacology, may reduce cardiovascular events in patients with atherosclerotic disease.

## Introduction

Cardiovascular disease (CVD) has been the leading cause of mortality in the United States of America (USA) every year since 1900, except 1918 (World War I) [1]. Despite an almost 30% decrease in CVD-related mortality over the last decade, it remains the leading cause of mortality in the USA, accounting for over 1.4 million deaths annually. This equates to approximately 2200 deaths per day or 1 every 40 s. Globally, CVD accounts for over 17.3 million deaths or 31% of total global mortality [2]. Current estimates state that 85.6 million people in the USA have some form of CVD, and by 2030, it is expected that approximately 44% of the US population will have CVD [1]. Total direct and indirect costs of CVD in the USA are over $316 billion annually and are expected to grow to over $918 billion by 2030. Direct costs are mainly composed of hospital admissions, with over 69 million physician office visits and over 4.3 million emergency department visits annually [1].

Much of the etiology of CVD is due to atherosclerosis and its thrombotic complications. Arterial beds most commonly impacted include the coronary, cerebral, and major peripheral arteries, including the renal, mesenteric, and lower extremity arteries. As atherosclerosis is a systemic disease, it rarely impacts a single arterial bed, even if symptoms have not developed [3]. It is known that patients with a history of myocardial infarction (MI) are not only at risk of recurrent MI but also at higher risk of stroke than the general population [3–5]. Similarly, patients with a history of stroke are not only at a higher risk of recurrent stroke but also at a higher risk of MI than the general population [3, 6]. Patients with peripheral arterial disease (PAD) also have higher risk of MI and stroke than those without PAD [3, 7].

Due to the central role of platelets in the pathophysiology of arterial thrombosis, antiplatelet therapy is critical for the acute and chronic treatment of patients with atherosclerotic disease, regardless of the arterial bed impacted. In the coronary vasculature, dual antiplatelet therapy with clopidogrel and aspirin has been the standard of care in the management of patients with acute coronary syndrome (ACS) since the results of the Clopidogrel in Unstable angina to prevent Recurrent Events (CURE) trial in 2001 [4, 5, 8]. In the cerebral and peripheral vascular beds, antiplatelet therapy has not advanced significantly beyond aspirin [6, 7].

The PARTHENON program, which was sponsored by AstraZeneca, is a series of completed and ongoing phase III clinical trials investigating the efficacy and safety of ticagrelor in atherosclerotic CVD in comparison with established antiplatelet therapy or placebo. The overall aim of the program is to determine if more potent antiplatelet therapy, with some different pharmacology, may reduce cardiovascular (CV) events in patients with atherosclerotic disease. The trials included in the PARTHENON program are listed in Table 1.

**Table 1:** Clinical trials included in the PARTHENON program

Trial acronym	Full trial name	Size (*n*)	Patient population
PLATO	The Study of Platelet Inhibition and Patient Outcomes	18,624	Acute coronary syndrome
PEGASUS–TIMI 54	Prevention of Cardiovascular Events in Patients with Prior Heart Attack Using Ticagrelor Compared to Placebo on a Background of Aspirin–Thrombolysis in Myocardial Infarction 54	21,162	At least 1 year post-myocardial infarction
SOCRATES	Acute Stroke or Transient Ischaemic Attack Treated with Aspirin or Ticagrelor and Patient Outcomes	13,199	Acute ischemic stroke
EUCLID	Examining Use of tiCagreLor In paD	13,885	Peripheral artery disease
THEMIS	Effect of Ticagrelor on Health Outcomes in Diabetes Mellitus Patients Intervention Study	~ 19,000	Type 2 diabetes mellitus with known coronary artery disease

## Oral Antiplatelet Therapy

### Aspirin

Aspirin, or acetylsalicylic acid, provides its antiplatelet effect by irreversibly inhibiting platelet cyclooxygenase (COX). This is accomplished by aspirin acetylating a specific hydroxyl group of serine 530 on COX-1 enzyme, which inhibits the binding of arachidonic acid [9]. Therefore, arachidonic acid cannot be converted to prostaglandin G_2_, which leads to reduced downstream production of thromboxane A2 [10, 11]. Aspirin has demonstrated benefit in reducing thrombotic events in patients with atherosclerosis, regardless of the vascular bed [12]. Chronic low-dose aspirin (75 to 100 mg daily) has demonstrated similar efficacy and reduced bleeding compared with chronic higher-dose aspirin (200 to 325 mg daily) and is typically preferred for long-term therapy [3, 4, 9, 12, 13].

## P2Y_12_ Receptor Inhibitors

### Clopidogrel

Clopidogrel is an orally administered, selective, irreversible inhibitor of the platelet P2Y_12_ receptor [14, 15]. Clopidogrel is a thienopyridine prodrug that requires a two-step hepatic activation via several cytochrome P450 (CYP) enzymes [16]. While several CYP enzymes are involved in the conversion of clopidogrel to its active metabolite, the largest contributor is CYP2C19, which accounts for over 50% of active compound creation [15, 17]. The active metabolite of clopidogrel is responsible for binding to the P2Y_12_ receptor, which leads to platelet inhibition. The active metabolite of clopidogrel has a reactive thiol group, which forms a disulfide bridge with the cysteine residues on the P2Y_12_ receptor, creating an irreversible inhibition of the P2Y_12_ receptor for the life of the platelet [18]. This ultimately leads to prevention of adenosine diphosphate (ADP)-mediated platelet activation and aggregation [15, 18].

Clopidogrel was initially evaluated in comparison to aspirin in the CAPRIE trial (Clopidogrel versus Aspirin in Patients at Risk of Ischaemic Events) [19]. The CAPRIE trial included 19,185 patients with a history of stroke, MI, or PAD and followed for a mean of 1.9 years. While there was a significant 8.7% relative reduction with the use of clopidogrel over aspirin in the primary endpoint of MI, stroke, or vascular death, the absolute reduction was relatively small (5.3 vs. 5.8%; *P* = 0.043). Interestingly, patients enrolled with a history of PAD demonstrated the greatest benefit from clopidogrel over aspirin, with a 23.8% relative reduction in the primary endpoint (*P* = 0.003).

Clopidogrel in combination with aspirin was compared to aspirin alone in the CURE trial (*n* = 12,562) [8]. In these patients with non-ST-elevation ACS, clopidogrel and aspirin significantly reduced the incidence of the primary endpoint of MI, stroke, or CV death compared to aspirin alone (9.3 vs. 11.4%; *P* < 0.001). There was also an increase in major bleeding with dual antiplatelet therapy compared to aspirin alone (3.7 vs. 2.7%; *P* = 0.001). The results of the CURE trial created the basis for dual antiplatelet therapy to become the standard of care in patients with ACS.

Despite the widespread use of clopidogrel, there continues to be a significant rate of recurrent CV events [8, 20]. These events are potentially explained by issues related to clopidogrel, including variability in antiplatelet response in up to 40% of patients, pharmacogenomic influences, and drug interactions [21–24]. The prescribing information for clopidogrel was updated in 2016 to include a warning on the potential impact of CYP2C19 polymorphisms on clopidogrel pharmacokinetics and clinical response at the request of the US Food and Drug Administration (FDA) [80]. While tests are available to identify a patient’s CYP2C19 genotype, current treatment guidelines do not recommend routine testing for the polymorphism.

### Prasugrel

Prasugrel, a third-generation P2Y_12_ receptor inhibitor, overcame a number of limitations of clopidogrel but has a similar thienopyridine chemical structure [25]. Prasugrel is also a prodrug that requires hepatic conversion to its active compound, but this is a single step with multiple enzymes assisting in the conversion. Therefore, conversion of prasugrel to the active compound is much more efficient and successful compared with clopidogrel. Prasugrel also has a more rapid onset of action, a prolonged duration of antiplatelet effects, and a more consistent antiplatelet activity compared with clopidogrel [26, 27]. Prasugrel was compared to clopidogrel in patients with ACS undergoing percutaneous coronary intervention (PCI) in the TRITON–TIMI 38 trial (Trial to Assess Improvement in Therapeutic Outcomes by Optimizing Platelet Inhibition with Prasugrel–Thrombolysis in Myocardial Infarction) [28]. All patients (*n* = 13,608) also received aspirin. After approximately 12 months, patients receiving prasugrel demonstrated a significant reduction in the primary endpoint of MI, stroke, and CV mortality (9.9 vs. 12.1%; *P* < 0.001). However, this was at the expense of significantly more non-coronary artery bypass grafting (CABG) major bleeding, life-threatening, and fatal bleeding. Bleeding was specifically higher in patients with a history of transient ischemic attacks (TIAs) or stroke, aged 75 years or older, or with a body weight < 60 kg (132 lbs). Conversely, no differences in efficacy (MI, stroke, or CV death) or safety (major bleeding) were found between prasugrel and clopidogrel (with aspirin) when the agents were evaluated in ACS patients not treated with PCI in the TRILOGY ACS trial (Targeted Platelet Inhibition to Clarify the Optimal Strategy to Medically Manage Acute Coronary Syndromes) [29]. Data with prasugrel in other vascular beds are currently lacking.

### Ticagrelor

Ticagrelor represents the first oral, direct-acting, reversible P2Y_12_ receptor inhibitor. Ticagrelor belongs to the chemical class of cyclopentyltriazolopyrimidines, which was developed from the chemical structure of a natural inhibitor of the P2Y_12_ receptor, adenosine triphosphate (ATP) [30]. Compared with the thienopyridine P2Y_12_ inhibitors mentioned above, ticagrelor is not a prodrug and does not require hepatic activation prior to providing its antiplatelet activity, nor is it influenced by CYP2C19 genetic variants [31]. This likely contributes to the lack of significant variability in antiplatelet activity, especially compared with clopidogrel. While the thienopyridines clopidogrel and prasugrel bind irreversibly to the P2Y_12_ receptor for the life of the platelet, ticagrelor has demonstrated both reversible and non-competitive binding to the P2Y_12_ receptor at a site that is different to that of the endogenous agonist ADP [32]. Therefore, the antiplatelet effects of ticagrelor also dissipate more quickly than clopidogrel or prasugrel once the drug is discontinued, which may be associated with faster restoration of platelet function.

In addition to its antiplatelet effects, ticagrelor has also demonstrated the ability to increase adenosine concentrations. This most likely occurs through inhibition of the sodium-independent equilibrative nucleoside transporter 1 (ENT-1) [33–35]. Erythrocyte ENT-1 is responsible for uptake of adenosine into the cell, where it is metabolized by multiple mechanisms. The ability of ticagrelor to inhibit adenosine’s uptake via ENT-1, and therefore increase adenosine’s systemic exposure, is likely due to the similar chemical structure of the two molecules, since ticagrelor was developed through multiple chemical modifications of ATP [35]. The quantifiable impact of the increased adenosine exposure is not fully understood but may provide clinical advantages and disadvantages to ticagrelor. Studies have demonstrated that ticagrelor can augment both endogenous and exogenous adenosine-induced coronary blood flow, which may provide a clinical advantage by producing improved perfusion in ischemic myocardium [33, 34]. Increased adenosine exposure may also explain some of the unique adverse effects observed with ticagrelor, (e.g., dyspnea, ventricular pauses, and gout) that are not typically seen with the thienopyridine P2Y_12_ inhibitors [36, 37].

Ticagrelor is rapidly absorbed, reaching peak concentrations in 2 to 3 h after multiple twice-daily dosing [30]. Ticagrelor is principally metabolized via the CYP3A4 and 3A5 enzymes. The active metabolite of ticagrelor, AR-C124910XX, represents about one third of ticagrelor metabolism. The mean elimination half-life of ticagrelor and that of its active metabolite are approximately 6.7 to 9.1 h and 7.5 to 12.4 h, respectively. Renal elimination of ticagrelor and the active metabolite is minor and not impacted by changes in renal function [30].

Numerous pharmacodynamic studies have demonstrated that ticagrelor provides rapid and potent platelet inhibition [30]. Compared with patients receiving a 600-mg loading dose of clopidogrel, followed by 75 mg daily, a 180-mg loading dose of ticagrelor, followed by 90 mg twice daily, provided significantly faster and more potent inhibition of platelet aggregation (IPA) [38]. Within 0.5 h, IPA with 20 μmol/L ADP was already significantly greater with ticagrelor compared with clopidogrel (41 vs. 8%; *P* < 0.0001). By 2 h, IPA was 88% with ticagrelor compared with 38% with clopidogrel (*P* < 0.0001) [38]. Ticagrelor was also shown to provide significant platelet inhibition in patients who were not considered responders to clopidogrel [39]. In a study of 41 clopidogrel non-responders, platelet aggregation dropped from 59% with clopidogrel down to 35% when these patients were switched to ticagrelor (*P* < 0.001). These results show that ticagrelor provides more potent and faster platelet inhibition compared with clopidogrel, regardless of the patient response to clopidogrel [39].

## The PARTHENON Program

Compared to thienopyridines, ticagrelor does not need hepatic activation, has a rapid onset of antiplatelet activity, potent platelet activity, consistent antiplatelet activity, reversible receptor binding, and a more rapid restoration of platelet function. Ticagrelor also has the potential to possibly improve coronary perfusion through increased adenosine exposure. Due to these pharmacologic advantages of ticagrelor over thienopyridines, a clinical trial program was developed to evaluate the role of ticagrelor in different types of atherosclerotic disease.

### PLATO—Acute Coronary Syndrome

Dual antiplatelet therapy with aspirin and the P2Y_12_ receptor antagonist clopidogrel has demonstrated a significant benefit over aspirin alone in patients with non-ST-segment elevation (NSTE) ACS in the CURE trial in 2001 [8]. Since these results, dual antiplatelet therapy has been considered standard of care for patients with ACS and has been incorporated into current treatment guidelines [3–5]. Despite the use of clopidogrel-based dual antiplatelet therapy, patients still have event rates of CV death, MI, or stroke of over 10% at 1 year [8, 28, 40].

The PLATO trial represents the phase III clinical trial that evaluated the efficacy and safety of ticagrelor compared with clopidogrel in patients with ACS [40]. Patients (*n* = 18,624) in the PLATO trial who presented within 24 h of an ACS event (NSTE ACS or ST-segment elevation MI [STEMI]) were randomized in a double-blinded fashion to a ticagrelor loading dose 180 mg, followed by 90 mg twice daily, or a clopidogrel loading dose of 300 or 600 mg, followed by 75 mg daily, for at least 6 and up to 12 months. All patients also received aspirin therapy dosed at the investigators’ discretion. The primary efficacy end point of the trial was the composite of CV death, MI, and stroke.

There was a significant 16% relative reduction in the primary end point with the use of ticagrelor compared with clopidogrel (hazard ratio [HR] 0.84; 95% confidence interval [CI] 0.77–0.92) (Table 2) [40]. The benefit of ticagrelor over clopidogrel was evident within the first 30 days of treatment (4.8 vs. 5.4%; *P* = 0.045) and continued to increase from days 31 to 360 (5.3 vs. 6.6%; *P* < 0.001). Therefore, the benefit demonstrated with ticagrelor in the PLATO trial was not just due to early potent antiplatelet therapy but also to maintained potent antiplatelet therapy. Definite stent thrombosis was also significantly reduced with the use of ticagrelor compared with clopidogrel in patients undergoing PCI with stenting (HR 0.67; 95% CI 0.50–0.91; *P* = 0.009) [40].

**Table 2:** Efficacy and safety findings from the PLATO trial at 12 months [40]

Outcome (%)	Ticagrelor	Clopidogrel	HR (95% CI)	*P*-value
Efficacy				
Cardiovascular death, MI, or stroke	9.8	11.7	0.84 (0.77–0.92)	<0.001
Cardiovascular death	4.0	5.1	0.79 (0.69–0.91)	0.001
MI	5.8	6.9	0.84 (0.75–0.95)	0.005
Stroke	1.5	1.3	1.17 (0.91–1.52)	0.22
Death from any cause	4.5	5.9	0.78 (0.69–0.89)	<0.001
Stent thrombosis—definite	1.3	1.9	0.67 (0.50–0.91)	0.009
Stent thrombosis—definite or probable	2.2	2.9	0.75 (0.59–0.95)	0.02
Primary end point—invasive approach	8.9	10.6	0.84 (0.75–0.94)	0.003
Primary end point—ischemia-driven approach				
Safety				
PLATO total major bleeding	11.6	11.2	1.04 (0.95–1.13)	0.43
TIMI total major bleeding	7.9	7.7	1.03 (0.93–1.15)	0.57
PLATO non-CABG major bleeding	4.5	3.8	1.19 (1.02–1.38)	0.03
TIMI non-CABG major bleeding	2.8	2.2	1.25 (1.03–1.53)	0.03
Need for transfusion	8.9	8.9	1.00 (0.91–1.11)	0.96
Life-threatening bleeding	5.8	5.8	1.03 (0.90–1.16)	0.70
Intracranial bleeding	0.3	0.2	1.87 (0.98–3.58)	0.06
Fatal bleeding	0.3	0.3	0.87 (0.48–1.59)	0.66
PLATO major or minor bleeding	16.1	14.6	1.11 (1.03–1.20)	0.008
TIMI major or minor bleeding	11.4	10.9	1.05 (0.96–1.15)	0.33
Dyspnea—any	13.8	7.8	1.84 (1.68–2.02)	<0.001
Dyspnea—requiring stopping study drug	0.9	0.1	6.12 (3.41–11.01)	<0.001
Holter identified ventricular pauses ≥ 3 s at 7 days	5.8	3.6	NR	0.01
Holter identified ventricular pauses ≥ 3 s at 30 days	2.1	1.7	NR	0.52

*CAGB* coronary artery bypass graft, *CI* confidence interval, *HR* hazard ratio, *MI* myocardial infarction; *NR* not reported; *PLATO* platelet inhibition and patient outcomes, *TIMI* thrombolysis in myocardial infarction

Of the individual components of the composite primary end point, MI was significantly reduced by 16% with the use of ticagrelor compared with clopidogrel (HR 0.84; 95% CI 0.75 to 0.95) [40]. There was also a significant 21% reduction in the incidence of CV mortality (HR 0.79; 95% CI 0.69 to 0.91), which has rarely been demonstrated with an oral antiplatelet agent [41, 42]. It has yet to be determined if the reduction in CV death demonstrated with ticagrelor is due to its more potent antiplatelet effect compared with clopidogrel, improved adenosine-induced coronary perfusion, or both.

The benefit of ticagrelor over clopidogrel in reducing CV events in the PLATO trial was evident regardless of the management strategy. The magnitude of effect of ticagrelor was consistent between patients in whom an invasive strategy was planned (*n* = 13,408; HR 0.84; 95% CI 0.75 to 0.94) and those assigned to a non-invasive, ischemia-driven approach (*n* = 5216; HR 0.85; 95% CI 0.73 to 1.00) [42, 43]. The results were similar for the subgroup of patients undergoing primary PCI for STEMI (*n* = 7544; HR 0.87; 95% CI 0.75 to 1.01; *P* = 0.07), and in patients who underwent CABG surgery during the trial (planned or not) and who received their last dose of study drug within 7 days before surgery (*n* = 1899; HR 0.84; 95% CI 0.60 to 1.16; *P* = 0.29) [44, 45].

The chronic dose of aspirin was discovered to have an important influence on clinical outcome in the PLATO trial. In a subanalysis of the PLATO trial, the benefit of ticagrelor appeared to be attenuated in patients enrolled in North America, specifically the USA [46]. Patients in the PLATO trial enrolled in the USA demonstrated a numerical increase in the primary end point with the use of ticagrelor compared with clopidogrel (11.9 vs. 9.5%; *P* = 0.1459), as well as each of the individual components of the composite end point. In further statistical analysis, it has been determined that this finding is likely due to the higher maintenance dose of aspirin used in the US compared with the rest of the world [46]. Patients enrolled in the USA were more likely to take a median maintenance aspirin dose of ≥ 300 mg/day (53.6%) compared with the rest of the world (1.7%). Those patients who received a maintenance dose of aspirin of ≥ 300 mg/day in the USA had an increase in risk of CV events with the use of ticagrelor compared with clopidogrel (HR 1.62; 95% CI 0.99 to 2.64), but a reduction in CV events if the maintenance dose of aspirin was ≤ 100 mg (HR 0.73; 95% CI 0.40 to 1.33). It had also been discovered that the effect of aspirin dose on CV outcomes was not just a phenomenon revealed in the USA. Patients in the rest of world also demonstrated an impact of aspirin dose on CV outcomes, with patients receiving a lower maintenance dose of aspirin having benefit with ticagrelor compared with clopidogrel (HR 0.78; 95% CI 0.69 to 0.87) that seemed to be lost with a higher maintenance dose of aspirin (HR 1.23; 95% CI 0.71 to 2.14). Based on these data, maintenance doses of aspirin 75 to 100 mg daily are recommended in order for ticagrelor to demonstrate benefits over clopidogrel, and higher chronic aspirin doses are contraindicated [47].

The incidence of total major bleeding using either the PLATO or Thrombolysis In Myocardial Infarction (TIMI) definition was not significantly increased with ticagrelor use compared with clopidogrel (Table 2) [40]. While total major bleeding was used as the primary safety end point in the PLATO trial, most antiplatelet therapy ACS trials use non-CABG major bleeding as the primary safety outcome due to the high rate of major bleeding seen in CABG surgery. When non-CABG major bleeding was evaluated, there was a significant increase for patients receiving ticagrelor compared with clopidogrel for PLATO major bleeding (HR 1.19; 95% CI 1.02 to 1.38; *P* = 0.03), as well as TIMI major bleeding (HR 1.25; 95% CI 1.03 to 1.16; *P* = 0.03). It should be noted that the incidences of life-threatening bleeding (5.8% in both groups) and fatal bleeding (0.3% in both groups) were not increased with the use of ticagrelor compared with clopidogrel [40].

Based on the different chemical structure of ticagrelor compared with the traditional thienopyridine P2Y_12_ inhibitors, a number of unique side effects have been reported that have not been traditionally reported with the thienopyridine class of P2Y_12_ inhibitors (Table 2) [40]. In the PLATO trial, patients randomized to ticagrelor had a significantly higher rate of reported dyspnea compared with those randomized to clopidogrel (13.8 vs. 7.8%; *P* < 0.001). Of the patients who reported dyspnea while receiving ticagrelor, 5.9% prematurely discontinued therapy compared with 1.6% of the clopidogrel patients who reported dyspnea (*P* < 0.001). The overall discontinuation rate due to dyspnea was 0.9% for ticagrelor and 0.1% for clopidogrel (*P* < 0.001). Most cases were judged to be mild to moderate in severity and occurred early in therapy, with resolution within 1 to 2 weeks of ticagrelor initiation [48]. In the PLATO trial, there was no change in pulmonary function demonstrated in a subset of patients who underwent pulmonary function testing (*n* = 199) with ticagrelor or clopidogrel [48]. Furthermore, patients with prior history of heart failure, chronic obstructive pulmonary disease, or other causes of dyspnea were not at higher risk of developing ticagrelor-related dyspnea.

Another side effect noted with the use of ticagrelor has been an increase in ventricular pauses of ≥ 3 s. In the 2908 patients in the PLATO trial who had a 7-day continuous electrocardiogram (ECG) recorder, pauses occurred in more patients receiving ticagrelor than clopidogrel (5.8 vs. 3.6%; *P* = 0.006) [36, 40]. At a follow-up ECG recording at 30 days, there was a similar proportion of patients with pauses (2.1 vs. 1.7%; *P* = 0.52). Most of the difference between the groups was in the incidence of sinoatrial node pauses. Importantly, there were no differences between the groups in the incidence of clinically reported bradycardia adverse events such as dizziness, syncope, pacemaker placement, or cardiac arrest [40]. It should be noted that patients at increased risk of a bradycardic event (known sick sinus syndrome, second- or third-degree atrioventricular conduction block, or previously documented syncope suspected to be due to bradycardia unless treated with a pacemaker) were excluded from the PLATO trial.

### PEGASUS–TIMI 54—Long-Term Dual Antiplatelet Therapy Following MI

The optimal duration of dual antiplatelet therapy has remained a question for many years. While studies have demonstrated the benefits of dual antiplatelet therapy for up to a year in patients with an ACS event, it has remained unknown if longer duration of dual antiplatelet therapy is beneficial [8, 28, 40]. Of the coronary events that occur each year, approximately one third are recurrent events [1]. In addition, registry data have demonstrated that the risk of recurrent events over a 4-year period is over 18% [49]. Therefore, it is clear that patient risk of recurrent events is an ongoing concern. An initial study of dual antiplatelet therapy with clopidogrel plus low-dose aspirin was compared with low-dose aspirin alone in patients with a history of CVD or multiple risk factors for CVD [50]. After 28 months of follow-up, clopidogrel-based dual antiplatelet therapy did not demonstrate a significant reduction in the primary end point of CV death, MI, or stroke compared with aspirin alone (6.8 vs. 7.3%; *P* = 0.22). However, a *post hoc* subgroup analysis in those patients with previous MI suggested a potential benefit in those receiving dual antiplatelet therapy [51].

The PEGASUS–TIMI 54 trial specifically evaluated patients at least 1 year post-MI and utilized ticagrelor-based dual antiplatelet therapy compared with low-dose aspirin [52]. In the trial, patients with a previous MI at least 1 year prior to study enrollment (*n* = 21,162) were randomized in a double-blinded fashion to ticagrelor 90 mg twice daily, ticagrelor 60 mg twice daily, or placebo. All patients also received low-dose aspirin during the trial. The primary efficacy end point was the composite of CV death, MI, or stroke. The primary safety end point was TIMI major bleeding.

After a median follow up of 33 months, patients receiving ticagrelor 90 mg twice daily demonstrated a significant reduction in the rate of the primary end point compared with those receiving placebo (HR 0.85; 95% CI 0.75 to 0.96; *P* = 0.008) (Table 3) [52]. Patients receiving ticagrelor 60 mg twice daily also demonstrated a significant reduction in the rate of the primary end point compared with those receiving placebo, and at a similar degree of magnitude to the 90-mg dose (HR 0.84; 95% CI 0.74 to 0.95; *P* = 0.004). Interestingly, the magnitude of benefit was fairly consistent across all components of the primary end point. In patients randomized to ticagrelor 90 mg twice daily compared with placebo, the HR for CV death was 0.87 (*P* = 0.15), MI 0.81 (*P* = 0.01), and stroke 0.82 (*P* = 0.14). In patients randomized to ticagrelor 60 mg twice daily compared with placebo, the HR for CV death was 0.83 (*P* = 0.07), MI 0.84 (*P* = 0.03), and stroke 0.75 (*P* = 0.03). Therefore, the 60-mg dose of ticagrelor provided consistent efficacy over placebo compared with the 90-mg dose [52].

**Table 3:** Efficacy and safety data from the PEGASUS–TIMI 54 trial [52]

Outcome (%)	Ticagrelor 60 mg bid	Ticagrelor 90 mg bid	Placebo	HR (95% CI) for 60 mg bid vs placebo	*P*-value	HR (95% CI) for 90 mg bid vs placebo	*P*-value
Efficacy							
CV death, MI, or stroke	7.77	7.85	9.04	0.84 (0.74–0.96)	0.004	0.85 (0.75–0.96)	0.008
CV death	2.86	2.94	3.39	0.83 (0.68–1.01)	0.07	0.87 (0.71–1.06)	0.15
MI	4.53	4.40	5.25	0.84 (0.72–0.98)	0.03	0.81 (0.69–0.95)	0.01
Stroke	1.47	1.61	1.94	0.75 (0.57–0.98)	0.03	0.82 (0.63–1.07)	0.14
Death from any cause	4.69	5.15	5.1	0.89 (0.76–1.04)	0.14	1.00 (0.86–1.16)	0.99
Safety							
TIMI major bleeding	2.30	2.60	1.06	2.32 (1.68–3.21)	<0.001	2.69 (1.96–3.70)	<0.001
Intracranial hemorrhage	0.61	0.56	0.47	1.33 (0.77–2.31)	0.31	1.44 (0.83–2.49)	0.19
Fatal bleeding	0.25	0.11	0.26	1.00 (0.44–2.27)	1.00	0.58 (0.22–1.54)	0.27
Dyspnea—any	15.84	18.93	6.38	2.81 (2.50–3.17)	<0.001	3.55 (3.16–3.98)	<0.001
Dyspnea—requiring stopping study drug	4.55	6.50	0.79	6.60 (4.50–8.15)	<0.001	8.89 (6.65–11.88)	<0.001
Renal events	3.43	3.30	2.89	1.17 (0.94–1.45)	0.15	1.17 (0.94–1.46)	0.15
Bradyarrhythmia	2.32	2.04	1.98	1.24 (0.96–1.61)	0.10	1.15 (0.88–1.50)	0.321
Gout	1.97	2.28	1.51	1.48 (1.10–2.00)	0.01	1.77 (1.32–2.37)	<0.001

*bid* twice daily, *CI* confidence interval, *CV* cardiovascular, *HR* hazard ratio, *MI* myocardial infarction, *TIMI* thrombolysis in myocardial infarction

As might be expected with long-term exposure to dual antiplatelet therapy, in this trial, major bleeding was significantly increased with both ticagrelor 90 mg twice daily (HR 2.69; 95% CI 1.96 to 3.70; *P* < 0.001) and ticagrelor 60 mg twice daily (HR 2.32; 95% CI 1.68 to 3.21; *P* < 0.001) compared with placebo (Table 3) [52]. There was also significantly more TIMI minor bleeding and need for transfusion with either dose of ticagrelor when compared with placebo (Table 3). Despite the increased incidence of major bleeding with the use of ticagrelor, rates of intracranial bleeding and fatal bleeding were not different compared with placebo. Similar to the PLATO trial, patients randomized to ticagrelor demonstrated significantly more dyspnea and dyspnea-related drug discontinuation compared with those who received placebo (Table 3). While renal events and symptomatic bradycardia were not significantly different between the groups, there were more episodes of gout in patients randomized to either dose of ticagrelor. Based on the similar efficacy between the two doses of ticagrelor over placebo, and the numerically lower rates of bleeding and dyspnea with the lower dose of ticagrelor compared with the higher dose, ticagrelor 60 mg twice daily is the FDA-approved dose for patients who are at least 1 year post-MI [47].

### SOCRATES—Acute Stroke or TIA

In the USA, approximately 6.6 million people have a history of a stroke or TIA [1]. By 2030, this number is expected to grow by over 20% with an additional 3.4 million patients [53]. Of the nearly 800,000 strokes that occur each year, 23% are recurrent events [1]. Globally, 33 million people have a history of stroke or TIA, which accounts for 6.5 million deaths, or 11.8% of global mortality [54, 55]. Stroke is also the leading cause of long-term disability in the USA [56, 57]. After hospital discharge for stroke, less than half of patients go directly home, with the majority going to inpatient rehabilitation, a skilled nursing facility, or directly to a nursing home [58]. These costs account for approximately $33 billion annually [58].

Optimal treatment of acute stroke includes intravenous or intra-arterial fibrinolysis [59]. Unfortunately, most patients are not candidates for this therapy based on timing and other contraindications. Aspirin is the only other therapy proven to have a benefit in the treatment of acute stroke [59–61]. After initial therapy, antiplatelet therapy is currently recommended as the treatment of choice for prevention of further thrombotic events after an initial stroke or TIA [6]. Recurrent ischemic stroke and other adverse vascular events occur in 10 to 20% of patients in the 3 months following TIA or minor ischemic stroke, with one study suggesting that 69% of strokes occurred within 7 days of a TIA [51, 62, 63]. While dual antiplatelet therapy has not demonstrated a meaningful advancement in patients with ischemic stroke, the optimal choice of antiplatelet therapy remains unclear [64].

The SOCRATES trial evaluated if the use of ticagrelor might provide a better reduction in vascular events compared with the only other evaluated oral therapy in patients with non-hemorrhagic acute ischemic stroke or TIA in the high-risk 3-month period following an event [65]. Patients (*n* = 13,199) were randomized within 24 h of symptom onset in a double-blind, double-dummy fashion to ticagrelor 180 mg on day 1, followed by 90 mg twice daily, or aspirin 300 mg on day 1, followed by 100 mg once daily. Both therapies were continued through 90 days. Patients receiving fibrinolytic therapy for treatment of stroke were not included in the trial. The primary end point of the trial was the composite of stroke, MI, or death at 90 days.

Although there was an 11% reduction in the primary end point with the use of ticagrelor compared with aspirin in the SOCRATES trial, this difference did not achieve statistical significance (HR 0.89; 95% CI 0.78 to 1.01; *P* = 0.07) (Table 4) [65]. While there was no difference in the incidence of MI or CV death with the use of ticagrelor in this patient population, there was a reduction in the incidence of ischemic stroke (HR 0.87; 95% CI 0.76 to 1.00; *P* = 0.046) and all stroke (HR 0.86; 95% CI 0.75 to 0.99; *P* = 0.03) demonstrated with the use of ticagrelor compared with aspirin. Major bleeding, fatal bleeding, and intracranial bleeding were not increased with the use of ticagrelor compared with aspirin (Table 4). While there was a numerical increase in the incidence of major or minor bleeding with the use of ticagrelor compared with aspirin, this difference did not achieve statistical significance (HR 1.32; 95% CI 0.99 to 1.76; *P* = 0.06) [65].

**Table 4:** Efficacy and safety data from the SOCRATES trial [65]

Outcome (%)	Ticagrelor	Aspirin	HR (95% CI)	*P* value
Efficacy				
Death, MI, or stroke	6.7	7.5	0.89 (0.78–1.01)	0.07
CV death	1.0	0.9	1.18 (0.83–1.67)	0.36
MI	0.4	0.3	1.20 (0.67–2.14)	0.55
All stroke	5.9	6.8	0.86 (0.75–0.99)	0.03
Ischemic stroke	5.8	6.7	0.87 (0.76–1.00)	0.046
Fatal stroke	0.3	0.3	0.90 (0.77–1.06)	0.21
Safety				
Major bleeding	0.5	0.6	0.83 (0.52–1.34)	0.45
Fatal bleeding	0.1	0.1	NR	NR
Intracranial hemorrhage	0.2	0.3	0.68 (0.33–1.41)	0.30
Major or minor bleeding	1.6	1.2	1.32 (0.99–1.76)	0.06

*CI* confidence interval, *CV* cardiovascular, *HR* hazard ratio, *MI* myocardial infarction, *NR* not reported

### EUCLID—Peripheral Artery Disease

Approximately 8.5 million patients in the USA live with PAD [1]. Globally, this number grows to 202 million [66]. Most mortality directly associated with PAD is associated with amputation, which has a 1-year mortality rate of over 48% [67]. Patients with PAD in the USA also have significant resource utilization, with over 1.1 million physician office visits, 19,000 emergency department visits, and almost 300,000 hospital outpatient department visits [1]. One of the main issues with PAD is the fact that it is a marker for systemic atherosclerosis. If patients have symptomatic atherosclerosis in the peripheral arterial bed, they are likely to have atherosclerotic disease in their coronary and/or cerebral arterial beds as well. Patients with PAD have rates of MI and stroke that are two- to four-fold higher than the general population, as well as higher rates compared with patients with only atherosclerotic risk factors, but not symptomatic disease [7, 49, 68]. Another analysis also suggests higher rates of CV death, MI, or stroke in patients with PAD and an ACS event compared with those with an ACS event without PAD (19.3 vs. 10.2%; *P* < 0.001), possibly due to patients with PAD representing a group with more extensive systemic atherosclerotic disease [69].

Antiplatelet therapy with aspirin or clopidogrel is recommended for patients with PAD for prevention of CV death, MI, or stroke [7]. This recommendation is for patients with symptomatic PAD, as well as those with an ankle-brachial index (ABI) ≤ 0.9 without symptoms. While many clinicians are likely to use aspirin therapy first, treatment with the P2Y_12_ inhibitor clopidogrel may be more effective. In the CAPRIE trial, patients with symptomatic coronary, cerebral, or peripheral atherosclerotic disease demonstrated a relative reduction of 8.7% in CV death, MI, or stroke with the use of clopidogrel compared with aspirin (*P* = 0.043) [19]. Interestingly, this benefit was most profound (23.8%; *P* = 0.0028) in patients with PAD. Therefore, P2Y_12_ inhibitor therapy may be especially beneficial in this patient population.

Since clopidogrel may be considered optimal antiplatelet therapy for patients with PAD, the EUCLID trial evaluated if more potent P2Y_12_ inhibitor therapy with ticagrelor may reduce clinical outcomes compared with clopidogrel [70]. Patients (*n* = 13,885) in the EUCLID trial had symptomatic PAD defined as symptoms with an ABI ≤ 0.80 or prior lower extremity revascularization for symptomatic PAD at least 30 days ago [71]. Patients were randomized in a double-blinded fashion to clopidogrel 75 mg daily or ticagrelor 90 mg twice daily. The primary end point was the composite of CV death, MI, or ischemic stroke after 30 months of follow-up. The primary safety end point was TIMI major bleeding.

The results of the EUCLID trial demonstrated that there was no difference in the primary composite end point after a mean of 30 months of treatment with ticagrelor compared with clopidogrel (HR 1.02; 95% CI 0.92 to 1.13) (Table 5). While there was also no difference between the individual end points of CV death or MI, there was a significant reduction in the incidence of ischemic stroke with the use of ticagrelor compared with clopidogrel (HR 0.78; 95% CI 0.62 to 0.98) (Table 5). Other secondary outcomes such as hospitalization for acute limb ischemia or the need for lower-limb revascularization were also not different between the groups (Table 5). Interestingly, patients with a known history of coronary or carotid revascularization (*n* = 3815) and those with a history of stent implantation (*n* = 1968) did respond differently compared with those without these more advanced atherosclerotic disease features (*P* value for interaction 0.03 for both). Major TIMI bleeding was similar between the groups (HR 1.10; 95% CI 0.84 to 1.43), as well as intracranial, fatal, or TIMI minor bleeding (Table 5). There were higher rates of any bleeding and dyspnea with the use of ticagrelor compared with clopidogrel (Table 5).

**Table 5:** Efficacy and safety data from the EUCLID trial [71]

Outcome (%)	Ticagrelor	Clopidogrel	HR (95% CI)	*P* value
Efficacy				
CV death, MI, or ischemic stroke	10.8	10.6	1.02 (0.92–1.13)	0.65
CV death	5.2	4.9	1.07 (0.92–1.23)	0.40
MI	5.0	4.8	1.06 (0.91–1.23)	0.48
Ischemic stroke	1.9	2.4	0.78 (0.62–0.98)	0.03
Death from any cause	9.1	9.1	0.99 (0.89–1.11)	
Hospitalization for acute limb ischemia	1.7	1.7	1.03 (0.79–1.33)	0.85
Lower-limb revascularization	12.2	12.8	0.95 (0.87–1.05)	0.30
Safety				
TIMI major bleeding	1.6	1.6	1.10 (0.84–1.43)	0.49
Intracranial bleeding	0.5	0.5	1.06 (0.66–1.70)	0.82
Fatal bleeding	0.1	0.3	0.53 (0.25–1.13)	0.10
TIMI minor bleeding	1.2	1.0	1.32 (0.96–1.83)	0.09
Dyspnea	4.8	0.8	NR	<0.001
Any bleeding	2.4	1.6	NR	<0.001

*CI* confidence interval, *CV* cardiovascular, *HR* hazard ratio, *MI* myocardial infarction, *TIMI* thrombolysis in myocardial infarction, *NR* not reported

### THEMIS—Type 2 Diabetes Mellitus

Approximately 21.1 million adults in the USA have the diagnosis of diabetes mellitus (DM), with an estimated 8.1 million undiagnosed and over 80 million with prediabetes [1]. Type 2 DM (T2DM) accounts for 90 to 95% of all cases of DM in adults. Patients with T2DM have a two- to four-fold increase in risk of CVD compared with patients without DM [72, 73]. Furthermore, following MI, patients with DM have rates of mortality and recurrent events comparable to those without T2DM [74]. Current recommendations include the use of aspirin for primary prevention of CV events for patients with DM [75]. It has been well documented that patients with T2DM have higher platelet reactivity and a blunted response to aspirin. While the exact mechanism is unknown, it may be due to hyperglycemic-specific contributions such as COX-1 glycation and platelet insulin resistance [76]. In the PLATO trial, ticagrelor provided a consistent benefit over clopidogrel, regardless of whether patients did or did not have DM [77]. Ticagrelor has also demonstrated the ability to achieve better platelet inhibition compared with prasugrel in patients with DM with an ACS undergoing PCI [78].

The THEMIS trial (NCT01991795) is designed to evaluate the efficacy and safety of ticagrelor in patients aged 50 years or more with T2DM with known coronary artery disease (*n* ~ 19,000) but without a history of an MI or stroke [79]. Patients will be randomized to ticagrelor 60 mg twice daily or placebo in a double-blinded fashion. The primary end point will be the composite of CV death, MI, or stroke at 48 months. Results of the THEMIS trial are expected in late 2018 or early 2019.

## Conclusion

The PARTHENON trials to date have demonstrated superiority of ticagrelor in some trials and a neutral impact in others. The reason for these mixed results could comprise a number of factors, including the disease state or vascular bed evaluated, duration of follow-up, or the comparator agent. In the PLATO trial, ticagrelor demonstrated superiority to clopidogrel as part of dual antiplatelet therapy. These positive effects are likely due to the improved antiplatelet response and duration of therapy of 6 months to a year. The high-risk nature of patients with ACS also likely contributed to the separation in event rates. The potential increase in coronary perfusion from increased adenosine exposure also could have contributed to the reduced CV mortality in the acute setting. In the PEGASUS trial, there was also a superiority effect of ticagrelor and aspirin over aspirin alone. The absolute benefit was not as great as that demonstrated in the PLATO trial. Since most patients were almost 2 years from their index MI, these were not acute patients and had more stable disease. Therefore, the benefit of more potent antiplatelet effect was evident, but the acute benefit of potential improved coronary perfusion was likely absent.

In the SOCRATES trial, ticagrelor was neutral compared to aspirin in impacting the primary endpoint of stroke, MI, and death at 90 days. The 90-day time frame is the period for the highest rate for recurrent stroke in acute stroke trials. While ticagrelor did significantly reduce recurrent stroke at 90 days, this is not likely sufficient follow-up for reducing MI or CV death in patients without symptomatic cardiac disease. Therefore, the composite endpoint was not well matched for a 90-day endpoint. Approximately one third of patients were already taking aspirin at the time of their acute stroke. Therefore, these patients may already have a lack of response to antiplatelet therapy and/or a non-ischemic etiology that resembles a TIA. Ticagrelor also demonstrated a neutral effect compared to clopidogrel in the EUCLID trial. While patients with PAD do have MI, stroke, and CV death, it is not as acute a risk as in the setting of ACS. Only 29% of patients had a history of coronary artery disease. Since the comparison was clopidogrel instead of aspirin, the ability of the more potent antiplatelet therapy with ticagrelor may not have been able to produce a reduction in events in this lower-risk patient population. As mentioned previously, patients with a history of coronary or carotid revascularization, and those with stent implantation, responded more favorably to ticagrelor compared to clopidogrel.

Atherosclerosis is a systemic disease impacting mainly the coronary, cerebral, and major peripheral arteries. The thrombotic nature of this disease contributes to significant morbidity and mortality. While antiplatelet therapies are often employed for the treatment and prevention of CV events in these patients, the optimal therapy in each vascular bed remains unknown. The PARTHENON program is a broad clinical development program that is ongoing and will continue to test ticagrelor across a wide spectrum of patients with atherosclerosis. These studies will inform clinical practice on the role of ticagrelor in these patients. As with any antiplatelet therapy, these benefits must be weighed against adverse effects such as bleeding and dyspnea.

## References

[1] Mozaffarian D, Benjamin EJ, Go AS, Arnett DK, Blaha MJ, Cushman M (2016). Heart disease and stroke statistics-2016 update: a report from the American Heart Association. Circulation.

[2] Roth GA, Forouzanfar MH, Moran AE, Barber R, Nguyen G, Feigin VL (2015). Demographic and epidemiologic drivers of global cardiovascular mortality. N Engl J Med.

[3] Smith SC, Benjamin EJ, Bonow RO, Braun LT, Creager MA, Franklin BA (2011). AHA/ACCF secondary prevention and risk reduction therapy for patients with coronary and other atherosclerotic vascular disease: 2011 update: a guideline from the American Heart Association and American College of Cardiology Foundation. Circulation.

[4] O'Gara PT, Kushner FG, Ascheim DD, Casey DE, Chung MK, de Lemos JA (2013). 2013 ACCF/AHA guideline for the management of ST-elevation myocardial infarction: a report of the American College of Cardiology Foundation/American Heart Association task force on practice guidelines. Circulation.

[5] Amsterdam EA, Wenger NK, Brindis RG, Casey DE, Ganiats TG, Holmes DR (2014). 2014 AHA/ACC guideline for the management of patients with non-ST-elevation acute coronary syndromes: a report of the American College of Cardiology/American Heart Association task force on practice guidelines. Circulation.

[6] Kernan WN, Ovbiagele B, Black HR, Bravata DM, Chimowitz MI, Ezekowitz MD (2014). Guidelines for the prevention of stroke in patients with stroke and transient ischemic attack: a guideline for healthcare professionals from the American Heart Association/American Stroke Association. Stroke.

[7] Anderson JL, Halperin JL, Albert NM, Bozkurt B, Brindis RG, Curtis LH (2013). Management of patients with peripheral artery disease (compilation of 2005 and 2011 ACCF/AHA guideline recommendations): a report of the American College of Cardiology Foundation/American Heart Association task force on practice guidelines. Circulation.

[8] Yusuf S, Zhao F, Mehta SR, Chrolavicius S, Tognoni G, Fox KK (2001). Effects of clopidogrel in addition to aspirin in patients with acute coronary syndromes without ST-segment elevation. N Engl J Med.

[9] Vane JR, Botting RM (2003). The mechanism of action of aspirin. Thromb Res.

[10] Roth GJ, Majerus PW (1975). The mechanism of the effect of aspirin on human platelets. I. Acetylation of a particulate fraction protein. J Clin Invest.

[11] Burch JW, Stanford N, Majerus PW (1978). Inhibition of platelet prostaglandin synthetase by oral aspirin. J Clin Invest.

[12] Antithrombotic Trialists’ Collaboration (2002). Collaborative meta-analysis of randomised trials of antiplatelet therapy for prevention of death, myocardial infarction, and stroke in high risk patients. BMJ.

[13] Mehta SR, Tanguay JF, Eikelboom JW, Jolly SS, Joyner CD, Granger CB (2010). Double-dose versus standard-dose clopidogrel and high-dose versus low-dose aspirin in individuals undergoing percutaneous coronary intervention for acute coronary syndromes (CURRENT-OASIS 7): a randomised factorial trial. Lancet.

[14] Dorsam RT, Kunapuli SP (2004). Central role of the P2Y12 receptor in platelet activation. J Clin Invest.

[15] Jiang XL, Samant S, Lesko LJ, Schmidt S (2015). Clinical pharmacokinetics and pharmacodynamics of clopidogrel. Clin Pharmacokinet.

[16] Farid NA, Payne CD, Ernest CS, Li YG, Winters KJ, Salazar DE (2008). Prasugrel, a new thienopyridine antiplatelet drug, weakly inhibits cytochrome P450 2B6 in humans. J Clin Pharmacol.

[17] Kazui M, Nishiya Y, Ishizuka T, Hagihara K, Farid NA, Okazaki O (2010). Identification of the human cytochrome P450 enzymes involved in the two oxidative steps in the bioactivation of clopidogrel to its pharmacologically active metabolite. Drug Metab Dispos.

[18] Sangkuhl K, Klein TE, Altman RB (2010). Clopidogrel pathway. Pharmacogenet Genomics.

[19] CAPRIE Steering Committee (1996). A randomised, blinded, trial of clopidogrel versus aspirin in patients at risk of ischaemic events (CAPRIE). Lancet.

[20] Mehta SR, Yusuf S, Peters RJ, Bertrand ME, Lewis BS, Natarajan MK (2001). Effects of pretreatment with clopidogrel and aspirin followed by long-term therapy in patients undergoing percutaneous coronary intervention: the PCI-CURE study. Lancet.

[21] O'Donoghue ML, Braunwald E, Antman EM, Murphy SA, Bates ER, Rozenman Y (2009). Pharmacodynamic effect and clinical efficacy of clopidogrel and prasugrel with or without a proton-pump inhibitor: an analysis of two randomised trials. Lancet.

[22] Oliphant CS, Trevarrow BJ, Dobesh PP (2016). Clopidogrel response variability: review of the literature and practical considerations. J Pharm Pract.

[23] Tantry US, Bonello L, Aradi D, Price MJ, Jeong YH, Angiolillo DJ (2013). Consensus and update on the definition of on-treatment platelet reactivity to adenosine diphosphate associated with ischemia and bleeding. J Am Coll Cardiol.

[24] Kim HS, Chang K, Koh YS, Park MW, Choi YS, Park CS (2013). CYP2C19 poor metabolizer is associated with clinical outcome of clopidogrel therapy in acute myocardial infarction but not stable angina. Circ Cardiovasc Genet.

[25] Dobesh PP (2009). Pharmacokinetics and pharmacodynamics of prasugrel, a thienopyridine P2Y12 inhibitor. Pharmacotherapy.

[26] Sugidachi A, Asai F, Ogawa T, Inoue T, Koike H (2000). The in vivo pharmacological profile of CS-747, a novel antiplatelet agent with platelet ADP receptor antagonist properties. Br J Pharmacol.

[27] Wallentin L, Varenhorst C, James S, Erlinge D, Braun OO, Jakubowski JA (2008). Prasugrel achieves greater and faster P2Y12receptor-mediated platelet inhibition than clopidogrel due to more efficient generation of its active metabolite in aspirin-treated patients with coronary artery disease. Eur Heart J.

[28] Wiviott SD, Braunwald E, McCabe CH, Montalescot G, Ruzyllo W, Gottlieb S (2007). Prasugrel versus clopidogrel in patients with acute coronary syndromes. N Engl J Med.

[29] Roe MT, Armstrong PW, Fox KAA, White HD, Prabhakaran D, Goodman SG (2012). Prasugrel versus clopidogrel for acute coronary syndromes without revascularization. N Engl J Med.

[30] Dobesh PP, Oestreich JH (2014). Ticagrelor: pharmacokinetics, pharmacodynamics, clinical efficacy, and safety. Pharmacotherapy.

[31] Wallentin L, James S, Storey RF, Armstrong M, Barratt BJ, Horrow J (2010). Effect of CYP2C19 and ABCB1 single nucleotide polymorphisms on outcomes of treatment with ticagrelor versus clopidogrel for acute coronary syndromes: a genetic substudy of the PLATO trial. Lancet.

[32] van Giezen JJ, Berntsson P, Zachrisson H, Bjorkman JA (2009). Comparison of ticagrelor and thienopyridine P2Y(12) binding characteristics and antithrombotic and bleeding effects in rat and dog models of thrombosis/hemostasis. Thromb Res.

[33] van Giezen JJ, Sidaway J, Glaves P, Kirk I, Bjorkman JA (2012). Ticagrelor inhibits adenosine uptake in vitro and enhances adenosine-mediated hyperemia responses in a canine model. J Cardiovasc Pharmacol Ther.

[34] Wittfeldt A, Emanuelsson H, Brandrup-Wognsen G, van Giezen JJ, Jonasson J, Nylander S (2013). Ticagrelor enhances adenosine-induced coronary vasodilatory responses in humans. J Am Coll Cardiol.

[35] Armstrong D, Summers C, Ewart L, Nylander S, Sidaway JE, van Giezen JJ (2014). Characterization of the adenosine pharmacology of ticagrelor reveals therapeutically relevant inhibition of equilibrative nucleoside transporter 1. J Cardiovasc Pharmacol Ther.

[36] Scirica BM, Cannon CP, Emanuelsson H, Michelson EL, Harrington RA, Husted S (2011). The incidence of bradyarrhythmias and clinical bradyarrhythmic events in patients with acute coronary syndromes treated with ticagrelor or clopidogrel in the PLATO (platelet inhibition and patient outcomes) trial: results of the continuous electrocardiographic assessment substudy. J Am Coll Cardiol.

[37] Camm AJ, Garratt CJ (1991). Adenosine and supraventricular tachycardia. N Engl J Med.

[38] Gurbel PA, Bliden KP, Butler K, Tantry US, Gesheff T, Wei C (2009). Randomized double-blind assessment of the ONSET and OFFSET of the antiplatelet effects of ticagrelor versus clopidogrel in patients with stable coronary artery disease: the ONSET/OFFSET study. Circulation.

[39] Gurbel PA, Bliden KP, Butler K, Antonino MJ, Wei C, Teng R (2010). Response to ticagrelor in clopidogrel nonresponders and responders and effect of switching therapies: the RESPOND study. Circulation.

[40] Wallentin L, Becker RC, Budaj A, Cannon CP, Emanuelsson H, Held C (2009). Ticagrelor versus clopidogrel in patients with acute coronary syndromes. N Engl J Med.

[41] Lewis HD, Davis JW, Archibald DG, Steinke WE, Smitherman TC, Doherty JE (1983). Protective effects of aspirin against acute myocardial infarction and death in men with unstable angina. Results of a veterans administration cooperative study. N Engl J Med.

[42] Cannon CP, Harrington RA, James S, Ardissino D, Becker RC, Emanuelsson H (2010). Comparison of ticagrelor with clopidogrel in patients with a planned invasive strategy for acute coronary syndromes (PLATO): a randomised double-blind study. Lancet.

[43] James SK, Roe MT, Cannon CP, Cornel JH, Horrow J, Husted S (2011). Ticagrelor versus clopidogrel in patients with acute coronary syndromes intended for non-invasive management: substudy from prospective randomised PLATelet inhibition and patient outcomes (PLATO) trial. BMJ.

[44] Steg PG, James S, Harrington RA, Ardissino D, Becker RC, Cannon CP (2010). Ticagrelor versus clopidogrel in patients with ST-elevation acute coronary syndromes intended for reperfusion with primary percutaneous coronary intervention: a platelet inhibition and patient outcomes (PLATO) trial subgroup analysis. Circulation.

[45] Held C, Asenblad N, Bassand JP, Becker RC, Cannon CP, Claeys MJ (2011). Ticagrelor versus clopidogrel in patients with acute coronary syndromes undergoing coronary artery bypass surgery: results from the PLATO (platelet inhibition and patient outcomes) trial. J Am Coll Cardiol.

[46] Mahaffey KW, Wojdyla DM, Carroll K, Becker RC, Storey RF, Angiolillo DJ (2011). Ticagrelor compared with clopidogrel by geographic region in the platelet inhibition and patient outcomes (PLATO) trial. Circulation.

[47] AstraZeneca. BRILINTA (ticagrelor) prescribing information. 2016:http://www.azpicentral.com/brilinta/brilinta.pdf. Accessed 23 June 2017.

[48] Storey RF, Becker RC, Harrington RA, Husted S, James SK, Cools F (2011). Characterization of dyspnoea in PLATO study patients treated with ticagrelor or clopidogrel and its association with clinical outcomes. Eur Heart J.

[49] Bhatt DL, Eagle KA, Ohman EM, Hirsch AT, Goto S, Mahoney EM (2010). Comparative determinants of 4-year cardiovascular event rates in stable outpatients at risk of or with atherothrombosis. JAMA.

[50] Bhatt DL, Fox KA, Hacke W, Berger PB, Black HR, Boden WE (2006). Clopidogrel and aspirin versus aspirin alone for the prevention of atherothrombotic events. N Engl J Med.

[51] Bhatt DL, Flather MD, Hacke W, Berger PB, Black HR, Boden WE (2007). Patients with prior myocardial infarction, stroke, or symptomatic peripheral arterial disease in the CHARISMA trial. J Am Coll Cardiol.

[52] Bonaca MP, Bhatt DL, Cohen M, Steg PG, Storey RF, Jensen EC (2015). Long-term use of ticagrelor in patients with prior myocardial infarction. N Engl J Med.

[53] Ovbiagele B, Goldstein LB, Higashida RT, Howard VJ, Johnston SC, Khavjou OA (2013). Forecasting the future of stroke in the United States: a policy statement from the American Heart Association and American Stroke Association. Stroke.

[54] Centers for Disease Control and Prevention (CDC). Prevalence and most common causes of disability among adults—United States, 2005. MMWR Morb Mortal Wkly Rep. 2009;58:421-6. 19407734

[55] Murray CJ, Atkinson C, Bhalla K, Birbeck G, Burstein R, Chou D (2013). The state of US health, 1990-2010: burden of diseases, injuries, and risk factors. JAMA.

[56] Buntin MB, Colla CH, Deb P, Sood N, Escarce JJ (2010). Medicare spending and outcomes after postacute care for stroke and hip fracture. Med Care.

[57] GBD 2013 Mortality and Causes of Death Collaborators. Global, regional, and national age-sex specific all-cause and cause-specific mortality for 240 causes of death, 1990–2013: a systematic analysis for the Global Burden of Disease Study 2013. Lancet. 2015;385:117–1. 10.1016/S0140-6736(14)61682-2 PMC4340604 25530442

[58] Feigin VL, Forouzanfar MH, Krishnamurthi R, Mensah GA, Connor M, Bennett DA (2014). Global and regional burden of stroke during 1990-2010: findings from the global burden of disease study 2010. Lancet.

[59] Jauch EC, Saver JL, Adams HP, Bruno A, Connors JJ, Demaerschalk BM (2013). Guidelines for the early management of patients with acute ischemic stroke: a guideline for healthcare professionals from the American Heart Association/American Stroke Association. Stroke.

[60] International Stroke Trial Collaborative Group (1997). The international stroke trial (IST): a randomised trial of aspirin, subcutaneous heparin, both, or neither among 19435 patients with acute ischaemic stroke. Lancet.

[61] CAST (Chinese Acute Stroke Trial) Collaborative Group (1997). CAST: randomised placebo-controlled trial of early aspirin use in 20,000 patients with acute ischaemic stroke. Lancet.

[62] Giles MF, Rothwell PM (2007). Risk of stroke early after transient ischaemic attack: a systematic review and meta-analysis. Lancet Neurol.

[63] Rothwell PM, Warlow CP (2005). Timing of TIAs preceding stroke: time window for prevention is very short. Neurology.

[64] Diener HC, Bogousslavsky J, Brass LM, Cimminiello C, Csiba L, Kaste M (2004). Aspirin and clopidogrel compared with clopidogrel alone after recent ischaemic stroke or transient ischaemic attack in high-risk patients (MATCH): randomised, double-blind, placebo-controlled trial. Lancet.

[65] Johnston SC, Amarenco P, Albers GW, Denison H, Easton JD, Evans SR (2016). Ticagrelor versus aspirin in acute stroke or transient ischemic attack. N Engl J Med.

[66] Fowkes FG, Rudan D, Rudan I, Aboyans V, Denenberg JO, McDermott MM (2013). Comparison of global estimates of prevalence and risk factors for peripheral artery disease in 2000 and 2010: a systematic review and analysis. Lancet.

[67] Jones WS, Patel MR, Dai D, Vemulapalli S, Subherwal S, Stafford J (2013). High mortality risks after major lower extremity amputation in Medicare patients with peripheral artery disease. Am Heart J.

[68] Steg PG, Bhatt DL, Wilson PW, D'Agostino R, Ohman EM, Rother J (2007). One-year cardiovascular event rates in outpatients with atherothrombosis. JAMA.

[69] Patel MR, Becker RC, Wojdyla DM, Emanuelsson H, Hiatt WR, Horrow J (2015). Cardiovascular events in acute coronary syndrome patients with peripheral arterial disease treated with ticagrelor compared with clopidogrel: data from the PLATO trial. Eur J Prev Cardiol.

[70] Berger JS, Katona BG, Jones WS, Patel MR, Norgren L, Baumgartner I (2016). Design and rationale for the effects of ticagrelor and clopidogrel in patients with peripheral artery disease (EUCLID) trial. Am Heart J.

[71] Hiatt WR, Fowkes FG, Heizer G, Berger JS, Baumgartner I, Held P (2017). Ticagrelor versus clopidogrel in symptomatic peripheral artery disease. N Engl J Med.

[72] Fox CS, Coady S, Sorlie PD, Levy D, Meigs JB, D'Agostino RB (2004). Trends in cardiovascular complications of diabetes. JAMA.

[73] Fox CS, Coady S, Sorlie PD, D'Agostino RB, Pencina MJ, Vasan RS (2007). Increasing cardiovascular disease burden due to diabetes mellitus: the Framingham heart study. Circulation.

[74] Miettinen H, Lehto S, Salomaa V, Mahonen M, Niemela M, Haffner SM (1998). Impact of diabetes on mortality after the first myocardial infarction. The FINMONICA myocardial infarction register study group. Diabetes Care.

[75] Fox CS, Golden SH, Anderson C, Bray GA, Burke LE, de Boer IH (2015). Update on prevention of cardiovascular disease in adults with type 2 diabetes mellitus in light of recent evidence: a scientific statement from the American Heart Association and the American Diabetes Association. Circulation.

[76] Dokken B (2008). The pathophysiology of cardiovascular disease and diabetes: beyond blood pressure and lipids. Diabetes Spectr.

[77] James S, Angiolillo DJ, Cornel JH, Erlinge D, Husted S, Kontny F (2010). Ticagrelor vs. clopidogrel in patients with acute coronary syndromes and diabetes: a substudy from the PLATelet inhibition and patient outcomes (PLATO) trial. Eur Heart J.

[78] Alexopoulos D, Vogiatzi C, Stavrou K, Vlassopoulou N, Perperis A, Pentara I (2015). Diabetes mellitus and platelet reactivity in patients under prasugrel or ticagrelor treatment: an observational study. Cardiovasc Diabetol.

[79] A study comparing cardiovascular effects of ticagrelor versus placebo in patients with type 2 diabetes mellitus (THEMIS). https://clinicaltrials.gov/ct2/show/NCT01991795. Accessed 23 June 2017.

[80] Bristol-Myers Squibb/Sanofi Pharmaceuticals Partnership Prescribing Information. PLAVIX (clopidogrel bisulfate) tablets. Prescribing information. 2016:http://www.accessdata.fda.gov/drugsatfda_docs/label/2010/020839s042lbl.pdf. Accessed 23 June 2017.

